# Biotechnological Applications of Polymeric Nanofiber Platforms Loaded with Diverse Bioactive Materials

**DOI:** 10.3390/polym13213734

**Published:** 2021-10-28

**Authors:** M. R. El-Aassar, Omar M. Ibrahim, Ziad H. Al-Oanzi

**Affiliations:** 1 Department of Chemistry, College of Science, Jouf University, Sakaka 75471, Saudi Arabia; 2 Polymer Materials Research Department, Advanced Technology and New Material Institute, City of Scientific Research and Technological Applications (SRTA-City), New Borg El-Arab City, Alexandria 21934, Egypt; 3 Department of Medicine, Division of Oncology, Washington University School of Medicine, St. Louis, MO 63110, USA; oibrahim@buffalo.edu; 4 Department of Clinical Laboratories Sciences, College of Applied Medical Sciences, Jouf University, Sakaka 75471, Saudi Arabia

**Keywords:** electrospinning, nanofibers, wound dressing, biomaterials, antibacterial

## Abstract

This review article highlights the critical research and formative works relating to nanofiber composites loaded with bioactive materials for diverse applications, and discusses the recent research on the use of electrospun nanofiber incorporating bioactive compounds such as essential oils, herbal bioactive components, plant extracts, and metallic nanoparticles. Inevitably, with the common advantages of bioactive components and polymer nanofibers, electrospun nanofibers containing bioactive components have attracted intense interests for their applications in biomedicine and cancer treatment. Many studies have only concentrated on the production and performance of electrospun nanofiber loaded with bioactive components; in this regard, the features of different types of electrospun nanofiber incorporating a wide variety of bioactive compounds and their developing trends are summarized and assessed in the present article, as is the feasible use of nanofiber technology to produce products on an industrial scale in different applications.

## 1. Introduction

### Electrospinning and Its History

Nanotechnology was initially put forward by Nobel laureate Richard P Feynman in his great lecture “There’s Plenty of Room at the Bottom” [1]. Nanotechnology can be defined as the technology of creating nanoparticles and materials or manufacturing devices within the size range of 1 to 100 nanometers (nm). Nanomaterials behave differently from the original materials they were produced from. Nanoscale materials include nanoparticles, nanofibers, nanowires, and nanotubes [2]. Nowadays, nanoscale ingredients exemplify actual and extensive opportunities for stimulating essential science and practical know-how. The importance of nanoparticles is owing to the fact that the optical and electronic performances of bulk materials can be changed at the nanometer scale [3]. The properties of materials can be altered on this nanoscale for two significant purposes. First, materials on the nanoscale have, comparatively, a greater surface area than the equivalent larger material or substance. As a result, materials can be made more chemically reactive, and the strength of electrical properties can be changed as well. Secondly, at the nanometer scale, quantum effects can initiate optical, electrical, and magnetic actions which are different from those in the same material at larger scales [4]. In this regard, nanofibers are of remarkable interest for industry due to their ability to expand production [5], simplicity of manufacture, and the ability to manipulate their structural, physical, and functional characteristics and features. Throughout the last decade, the electrostatic spinning technique has been the most popular method of producing fibrous non-woven materials with diameters ranging from the nanometer to micrometer size scale. With a characteristic surface area to mass ratio and controlled surface morphology, and owing to its other properties, nanofiber can offer many benefits in a wide range of technologies, applications and products such as water filtration [6], biological substances [7], and batteries [8].

In this review, nanofibers are categorized into one or more subclasses of nanotechnology. It is obvious that nanofibers fall into the category of nanostructural materials, which also includes nanorods and nanotubes. Unlike these, however, the bendable nature of nanofiber also allows for it to be enriched laterally with other extremely elastic elements in nano form. Study of the mechanical properties of nanofiber would fall under the heading of nanomechanics. To this point, the processing performances of nanofibers are miscellaneous, and comprise both top-down and the bottom-up methodologies [9].

## 2. Fabrication Methods for Creating Nanofibers

The purpose of our work is to discuss the recent research on the different preparation methods and uses of nanofibers, while highlighting the common advantages of electrospun polymeric nanofibers and the diverse bioactive components that they can be loaded with. Among the techniques successfully used to create polymeric nanofibers are drawing, template synthesis, phase separation, self-assembly, wet spinning, melt spinning, and the electrospinning technique [10,11], all of which are described in the coming section, with simple comparisons between most common techniques shown in Table 1. Among these techniques, electrospinning is the most widely used for producing electrospun fibers because of its practicality, operational simplicity, cost-effectiveness, efficiency, and versatility [12].

## 3. Electrospinning Technique

The electrospinning technique has several advantages compared to the other techniques. The greatest benefit of electrospinning is the possibility of scaling up, as it can be applied on the industrial level and not only on the lab scale. Electrospinning is a simple and straightforward technique for producing uniform nanofibers with diameters in the nano- to micrometer range, with continuous and controllable production of fiber morphology. Generally, the electrospinning technique has become the most used to create electrospun nanofibers. Electrospinning is a simple process, easy and relatively inexpensive to setup, and a versatile technique for creating fibers with average fiber diameters was within the nanometer scale, which produces nanofiber with a high specific surface areas and large structured pores [13]. According to some findings, it may be possible to expand the scale of this technology to mass production. The development of the electrospinning mechanism began in the 1930s with the production of nanofibers by Formhals Anton [14]. In the 1990s, many research groups, notably the Reneker group, suggested that electrospun nanofibers could be fabricated from different organic polymers using the technique that had been known as electrostatic spinning, now electrospinning [15]. Subsequently, Reneker et al. used natural and synthetic polymers to investigate the feasibility of producing nanofibers via electrospinning. Since the late 19th century, the electrospinning technique has attracted great interest, which was reflected in several research publications every year. Currently, only the electrospinning technique is used to manufacture nanometer diameters of polymer nanofibers. Electrospinning has been used to generate a variety of nanofibers for a wide range of consumer applications such as filtration, medical devices, aerospace, energy, and healthcare [16]. Nowadays, nanofibers provide amazing potential in medical areas such as wound dressings, diabetes, cancer, tissue engineering, and drug delivery systems [17]. In general, nanofibers for medical purposes are perhaps the most promising of all their industrial purposes, and reinforced nanocomposites have also played an important role in the medical field for several reasons. This is evidenced by the number of papers published annually in scientific journals in various sciences. Nanofibers provide many unique special properties, such increasing the surface area of conventional materials [18], this can allow increased interaction and bonding between the targeted material and nanofibers in many domains compared to bulk materials. Moreover, the unique properties of polymeric nanofibers, such as their flexibility in the presence of functional groups on surfaces, high mechanical properties, and controllable pore size, cannot be found in other structures. In addition, the nanofiber structure is characterized by a high porosity network and lightweight, with flexibility and design efficiency for specific physical–chemical surface functions [17]. All these properties and advantages make non-woven nanofibers suitable materials for widespread application [19]. A wide range of polymers, both natural and synthetic, as well as polymer blends and other composite materials can be spun into nanofibers. Polymer selection is essential to produce nanofibers with application-specific attributes. The ideal polymer for biomedical applications should be biocompatible, biodegradable, non-toxic, moderately hydrophilic, and possess appropriate mechanical strength. The source of polymers used in nanofiber production can vary from natural to synthetic polymers [20], each of which has different sets of advantages and disadvantages; the end use of the nanofibers dictates the exact type of polymer to be used. Table 2 shows a classification of the polymers most commonly used in biomedical applications.

Polymers, whether natural or synthetic, can be electrospun to suit different applications with improved properties. Naturally produced polymers (natural polymers) have been used extensively in both biological and therapeutic applications because of their biodegradability, biocompatibility, and biological characteristics. Materials derived from plants or animals, which are mostly made up of proteins or polysaccharides, can mimic the fibrillary structure of the original ECM and have similar architecture [37]. Biopolymers are essentially similar to these materials in that they consist of proteins or polysaccharides [38]. Polysaccharide polymers are made of monosaccharide units joined together via glycosidic linkages. The use of these polymers as biomaterials has become popular as new biological functions are identified for such materials. Biological polymers are either linear or branched, depending on whether their purpose is structure or storage [39]. Biodegradability and biocompatibility make biopolymers very promising materials in medical and research applications. Recently, new synthetic methods have been developed to modify biopolymers to overcome setbacks associated with their functionalities and activities. Biopolymer-based biomaterials used for biomedical applications can be classified into several categories: neutral (e.g., β-glucan, dextran, cellulose), acidic (e.g., hyaluronic acid, alginic acid,), basic (e.g., chitin, chitosan), or sulfated (e.g., heparin, heparan sulfate, chondroitin sulfate, dermatan sulfate, keratan sulfate) [40]. Synthetic polymers are favored over natural polymers in certain uses, as they can be tailored to develop fibers with the optimum mechanical and degradation properties [41,42,43,44,45,46]. They can be blended with other natural or synthetic polymers to modify their mechanical performance and to permit the sustained release of loaded drugs [47,48,49,50].

## 4. Process of Electrospinning and Setup

The basic components of an electrospinning setup consist mainly of four major components to accomplish the process of producing strong and soft fibers: (1) a high voltage DC power supply; (2) a syringe pump; (3) a metallic spinneret or die (generally a needle); and (4) a collector (a grounded conductor) [38]. A syringe pump with a controller and spinneret of small diameter is used for pumping polymer solution and controlling the injection rate. The most important component is a high voltage electrical source and flat or rotor metallic collector, which is grounded. A cross-linked viscous polymer solution is pumped out via a fine steel syringe needle, and a high voltage is applied to the polymer droplet at the spinneret. A drop of polymer solution extends into a thin jet in flight. As a result, the critical surface charge density will increase on the polymer surface when the spinneret is highly electrified and the charge density will increase on the surface; the charged collector is placed in the opposite position while maintaining the distance in order to collect the extruded fibers. The solution in the spinneret is extruded by overcoming the surface tension and forming a cone-shaped structure called a “Taylor cone”. The Taylor cone is formed due to the collector’s high charge density, and the solvent will rapidly evaporate from the polymer due to the rapid transition from the spindle to the collector [42]. Although the principle of electrospinning is very simple, the process itself can be extremely difficult because many factors may affect fiber diameter and the final network of nanofibers. Generally, these factors can be divided into three parametrical categories: solution, processing, and ambient parameters. Solution parameters include viscosity, polymer concentration, molecular weight, surface tension and electrical conductivity. Processing parameters involve feed (flow) rate, electric field strength, tip-to-collector distance, needle (tip) shape, and collector composition and geometry. Finally, ambient parameters include temperature, humidity, and airflow. Nanofiber diameter, surface morphology, mechanical properties, porosity, and pore size distribution depend greatly on these parameters [51,52,53].

The process relies on the proper selection of a suitable solvent to dissolve the polymer. Moreover, optimal flow rates determine the success of the electrospinning process, with slower flow rates recommended in order to provide enough time for polymer solution polarization. Rapid and higher flow rates result in the production of fibers with a thicker diameter and the presence of beads on the surface due to the solvent not drying before reaching the collector [54,55,56]. The viscosity of the polymer solution must not be too great, or the surface tension of the polymer solution too high; this can prevent jet formation or cause the Taylor cone to be too small for the polymer solution to drain freely from the syringe [43]. Regarding the applied voltage, it must be suitable to overcome the effect of the viscosity and surface tension of the polymer solution in order to permit the formation of smooth and uniform nanofibers and continuous pumping of the polymer solution from the syringe without effect [44]. Finally, the distance between the metallic needle and the ground collector should be large enough to evaporate the polymer solvent in a timely manner and produce the nanofibers; however, the very small distance between the metallic needle and the collector may lead to sparks between the electrodes [57,58,59,60,61,62,63,64].

## 5. Types of Electrospinning Techniques for the Creation of Nanofibers

There are quite a numbers of electrospinning techniques for producing polymeric nanofibers, some of which are explained in the coming sections.

### 5.1. Blend Electrospinning

Blend electrospinning is based on combining of a wide variety of natural and synthetic polymers with bioactive molecules such as drugs [46], antibiotics [47], essential oils [26], herbal substances [48], and inorganic materials [49], which are dissolved or dispersed in the polymeric solution, resulting in bioactive materials encapsulated within nanofibers and spread on the inside of the fibers [50]. Although this method is simple compared to other electrospinning methods, the organic solvent or co-solvents used in the blend solution can lead to a reduction insensitive biological behavior, loss of activity of the protein, or protein denaturation. On the other hand, a blend of bioactive molecules with a mixture of natural and synthetic polymer is challenging.

### 5.2. Melt Electrospinning

The melt electrospinning process is applied for the formation of nanofiber by using molten polymers and vacuum [51]. Melt electrospinning offers many advantages, including ecofriendliness, no requirement for organic solvent, and the generation of submicron fibers from molten polymers [52]. The key advantage of melt electrospinning is the highly reproducible production of electrospun fiber mats from polymer materials with very low manufacturing costs, and without any effect on the volume produced due to solvent evaporation [51]. On the other hand, although melt electrospinning was proposed about 30 years ago, research has been limited and is still in the early stages due to the complicated nature of its setup and its limitations compared to solution electrospinning in terms of processing temperatures and viscosity as well as ability to obtain fibers over 10 microns in diameter [53].

### 5.3. Coaxial Electrospinning

Coaxial electrospinning has been used to generate core–shell nanofibers that consists of two complementary polymeric liquid solutions in the core and the shell using a concentric spinneret. The general system for coaxial electrospinning of core–sheath fibers is based on the utilization of polymeric liquids for each core and sheath layer using two nozzles, which are coaxially placed together. The inner liquid (core nozzle) is excreted through the internal nozzle, and the shell liquid is pumped through the outside sheath nozzle [54]. The processing conditions are critical for successful operation; sheath solution viscosity, polymer concentration of the electrospinnable solution, conductivity and the flow rate of the shell solution should all be higher than those of the core solution. On the other hand, the interfacial tension between the core and sheath solutions must enable designing of the core liquid and blending of the polymeric solutions should not be allowed in order to permit the formation of a stable Taylor cone [55]. The main advantage of the coaxial electrospinning technique is to allow combination of electrospinnable with non-electrospinnable solutions in the form of polymer/polymer, polymer/inorganic, and inorganic/inorganic solutions to produce uniform core–shell fibers. This coaxial technique provides enormous potential for developing various applications.

### 5.4. Emulsion Electrospinning

Emulsion spinning is a simple technology which combines both blend and coaxial electrospinning with the emulsification approach, stabilized by surfactants such as Span 80, Tween 80, and Pluronics to produce core–shell nanofibers from insoluble and non-melting compounds [17]. This method is used for encapsulation of hydrophilic inorganic materials and proteins. The emulsion method is particularly well suited for processing two-phase or multiple phase polymers, for instance, hydrophilic and lipophilic solutions for the formation of emulsion phases. This means that the polymer solution must immiscible and typically stabilized by emulsification [36]. Generally, the advantages of emulsion electrospinning lie in the production of very small particles with sustained release, good bioactivity, and effective of encapsulation of compounds after delivery, which release out of the carrier very quickly [7].

## 6. Applications of Bioactive Material-Containing Nanofibers

Metabolic syndrome (MetS) is a collection of metabolic abnormalities caused by inflammation and oxidative stress that contribute to chronic illnesses including diabetes and cardiovascular disease [65]. Plant extracts are one of the most intriguing treatment alternatives for treating MetS because of their unique benefits such as anti-inflammatory and antioxidant capabilities [66]. Medicinal plants and their extracted components have been proven in many studies to have positive therapeutic benefits such as anti-inflammatory and antioxidant capabilities and have antibacterial and anti-cancer properties as well [67]. Inflammation is a localized physical condition in which a portion of the body responds to an infection or damage with swelling, redness, pain, and other symptoms. It is the positive host response that leads to the restoration of cellular homeostasis as well as tissue shape and function. Inflammation is divided into two types: acute inflammation, which is less severe and limited to a particular region, and chronic inflammation, which persists after the pathogen that caused the acute inflammation is destroyed or eliminated [68]. It subsequently develops into an autoimmune condition in which normal, healthy host cells are attacked, resulting in illness [68]. Rheumatoid arthritis and in rare instances cancer may develop as a result of chronic inflammation [69]. The systemic generation of TNF-α by macrophages, which stimulate the central innate immune response, is a hallmark of acute and chronic inflammation. The NFkB and COX-2 pathways are important players in the up regulation of inflammation. Without an inflammatory response, infections, wounds, and tissue damage cannot recover [70,71,72].

The innate and adaptive immune responses are two key components of the host defensive systems that mediate this response. The innate immune response is the body’s first reaction to a foreign material, which is subsequently processed by granulocytes, phagocytes, and other cells in the adaptive immune response. Adaptive immunity is targeted and aids in the elimination of infections at a later stage, as well as the formation of immunological memory. When a host with a functioning innate immune system comes into contact with external stimuli, inflammation typically starts within minutes. Innate immunity has a major role in inflammation [73]. In the inflammatory phase of wound healing, physiologically active mediators attract neutrophils, leukocytes, and monocytes to wound sites, where they phagocytose bacteria and foreign debris, resulting in the generation of reactive oxygen species (ROS) [74]. To maintain redox equilibrium, or the balance between free radicals and antioxidants, the cell’s antioxidant system plays a key role in scavenging these free radicals. Superoxide ion (O_2_^–^), hydrogen peroxide (H_2_O_2_), the hydroxyl radical, and other reactive oxygen derivatives are highly deadly and cause significant damage to protein, DNA, and lipids, interfering with normal cellular function [75]. As a result of oxidative phosphorylation, reactive oxygen species are generated in the cell. At the cellular level, ROS are continuously produced. However, since they are scavenged by different antioxidant processes, they are incapable of causing damage. Because excessive amounts of ROS may harm cells by oxidizing lipids and proteins, they are carefully regulated by ROS scavenging enzymes and small molecule antioxidants. In response to hypoxia or other stimuli, angiogenesis is the development of a new microvascular capillary network [76]. Angiogenesis is a process in which hypoxic endothelium and supporting cells secrete angiogenic substances that promote endothelial proliferation and new vessel sprouting [76]. For effective vascular construction, a vast number of pro-and anti-angiogenic factors must work in a coordinated and synergistic way to permit the complicated process of angiogenesis. The buildup of reactive oxygen species in damaged tissues causes substantial loss of autologous stem cells, growth factors, and nucleic acids, reducing their regeneration potential and causing wound healing to be delayed [77]. In diabetic individuals, for example, vascular cells are exposed to excessively high quantities of ROS signaling molecules, resulting in imbalanced signal pathways. Furthermore, oxidative stress is widely recognized as a contributor to the development of diabetes complications such as cardiovascular disease, nephropathy and retinopathy due to altered oxidative signaling (imbalance between free radicals and antioxidants), all of which are treatable and preventable [78]. The essential natural components contained in plant herbs are distinguished by having extremely efficient chemicals such as sugars, amino acids, alkaloids, vitamins, enzymes, flavonoids, and phenolic compounds, which are regarded as among the most significant applications in many medical disciplines. Before the introduction of synthetic medicinal plants, materials used in medical treatment were considerably safer to use and seldom included harmful chemicals. However, while separating or isolating the active material that includes it, poisonous compounds and impurities are introduced owing to isolation techniques which use chemicals and separated instruments to accompany the active ingredient, resulting in toxicity during treatment. Usually, upon extraction of active ingredients from medicinal plants, processing is needed to enhance various characteristics; in some cases, such as with Flavonoids, processing can introduce toxic byproducts [79,80,81,82,83,84,85,86,87,88,89,90].

L. Hench discovered natural bioactive compounds in 1970 as a consequence of his research into the capacity of some glasses to show reactions that enable them to attach to bone tissue [80]. In general, a substance’s essential activity dictates its particular response when it comes into touch with host tissues, resulting in cross-linking. This kind of contact has a significant beneficial impact on the human body’s biological reaction as well as on cellular activity. This was a starting point for the creation of novel biologically active materials, and was developed and utilized in therapeutic applications. Various methods including melt cooling, co-deposition and colloidal solution gel have been explored by several researchers to produce highly biocompatible bioactive materials with structural characteristics, along with attempts throughout the last decades to achieve such biomaterials [81]. Because research on these kinds of materials focuses on applications in solid tissues, it has progressed in two distinct directions: the first is based on an in vivo assessment of the interaction between bone tissue and biomaterials, which supported the hypothesis that hydroxyl forms the interlayer of apatite [80]. The other was studied in vitro using SBF (simulated bodily fluids) and lives cells [82]. Both methods give a clear picture of what happens when bioactive chemicals come into touch with living tissue. Even though bioactive materials are currently being developed at the level of bone tissue, research has demonstrated that they may be utilized in a variety of applications, including regenerative medicine, tissue engineering, wound care, gene therapy, drug delivery systems, and diagnostics [83]. Meanwhile, researchers are focusing on electrospinning (ES), which is thought to be the most feasible method for creating improved scaffolding for tissue engineering and the delivery of signaling molecules to replace sick or damaged tissue [67]. The treatment of skin wounds is one example of such an application. A wound is described as a loss of epithelial tissue integrity caused by physical, chemical, electrical, or thermal damage [67]. Hemostasis, inflammation, proliferation, and maturation are the four phases of cutaneous wound closure that overlap at the time of damage; a fibrin-rich clot develops to bridge severed arteries, restore homeostasis, and function as a temporary biologically active scaffold. Platelet lysis, which shows the presence of phagocytic leukocytes in the blood to cleanse the afflicted region of foreign particles, increases inflammation [74]. Excess pollutants, on the other hand, may cause further tissue damage as a result of the excessive generation of reactive oxygen species (ROS). Similar fatalities were recorded in earlier research on individuals with chronic wounds and cancer [84]. However, excessive deposition of fibrous scarring results from imbalanced mammalian wound healing; none of the existing scar therapies are effective enough, and molecular remedies face significant challenges. As a result, skin defects may be seen as a healthcare cost, and further research is required to develop ulcer preventive dressings that reduce the chances of poor organ function while also removing the financial burden of scar revision. It is worth mentioning that the skin’s immune system is a key player in the rebalancing process that occurs during self-healing. Another common technique for wound healing is the use of medicinal plants; submerging them in different kinds of nanoparticles is one of the most promising methods to improve their efficacy [85]. Nanomaterials have unique characteristics owing to their nanoscale and high surface area to volume ratio and medicinal plant nano-scaling may occur in combination with alteration of their physical and chemical properties, making drug release management in the target organ essential [86]. Nanoparticles, nano-emulsions, nanohydrogels, nanomembranes, and nanoparticle liposomes are among the biocompatible drug carriers utilized in controlled drug delivery systems; by offering a sustainable and regulated delivery system, these systems may minimize side effects and improve the effectiveness of different medicinal agents, and can also increase the rate of drug dissolution due to their surfactant characteristics [86]. The use of compounds present in wound healing, making these treatments with nanoparticles, or incorporating materials into such nanoparticles provides an opportunity to control their delivery to the affected side and can increase their chemical activity, thus obtained results in a variety of ways the therapeutic goal of which is to regulate the wound healing process [87].

Curcumin and mineral nanoparticles derived from plants are efficient in promoting angiogenesis by controlling growth factors [88]. The pace of re-epithelialization is another important element in wound healing. Natural compounds including emodin, fenugreek, curcumin, and tragacanth gum are examples of nanostructures that may promote collagen production and fibroblast proliferation, resulting in faster re-epithelialization. Although all of the produced herbal nanostructures were effective in wound mitigation, curcumin-based nanostructures were determined to be the strongest of the nanostructures, playing a key role in wound healing management at all stages [87]. In pharmacology, *Calendula officinalis* (*C. officinalis*) is often found in medical preparations that are used in skin treatments as a wound-healing aid and anti-inflammatory. Most of the chemicals in it are phenolic compounds, which are very essential (such as flavonoids and coumarin). Studies on the various medicinal properties of *C. officinalis*, including as an anti-inflammatory, antibacterial, antifungal and antioxidant, as well as its potential to promote angiogenesis, have been conducted on many occasions. Triterpenes have been shown to stimulate fibroblast migration and proliferation [88,89]. Additionally, other compounds were identified and characterized, demonstrating anti-inflammatory, anticancer, and antioxidant properties. Studies in living rats have shown that the application of *C. officinalis* to skin wounds speeds up the rate of wound healing and decreases the time required for epithelium breakdown, and promotes angiogenesis [90]. In research by Naeini et al. on *C. officinalis* gel at various concentrations and on wound incision collagen production and hydroxyproline content in rats, *C. officinalis* gel significantly increased both collagen synthesis and hydroxyproline content in the wound incisions [91]. Collagen synthesis in the 7% *C. officinalis* gel applied topically was considerably better than placebo and the control groups [91]. The therapeutic outcomes obtained for *C. officinalis* in treating ulcers and acute dermatitis after breast cancer radiation were previously shown to correlate with a concentration dosage [92,93]. Pilot research was conducted by Binic et al. that included 32 participants to study the impact of herbal medications on the healing process of venous leg ulcers free of infection. In the group which used the herbal preparations, after seven weeks the topical usage reduced the amount of ulcer surface area and reduced bacterial colonization, while the control group saw no changes. Ulcer surfaces treated with herbal items saw a 42.68% reduction, while the control group showed a 35.65% drop, which demonstrates the beneficial impact of *C. officinalis* on wound healing [92].

Aloe vera (AV), also known as Aloe barbadensis Miller, is the most frequently used herbal treatment for treating wounds. AV is a member of the Liliaceae family that is native to the tropics. The resulting finished products from the processing of fresh plant leaves include two products, known as “Aloe vera latex” and “Aloe vera gel”. AV gels are the most beneficial for the treatment of skin lesions, comprising a water-based solution (usually 99.5%) and a slightly alkaline (0.5–1%) component that consists of several biologically active components such as soluble sugars, non-starch polysaccharides, lignin, lipids, vitamins (B1, B2, B6, and C), enzymes (acid phosphatase, alkaline phosphatase, amylase, and lipase), salicylic acids, proteins, and minerals (sodium, calcium, magnesium, and potassium)[94]. Anti-inflammatory, antiseptic, and antibacterial qualities have all been reported to stem from the AV gel. It is also capable of stimulating fibroblast proliferation, collagen production, and angiogenesis, which all aid in rejuvenation. It is widely accepted that several polysaccharides (e.g., acemannan, mannose-6-phosphate, pectic acid, galactan, and glucomannan) and glycoproteins (e.g., lectins) in the leaf pulp are critical to wound healing. This claim is supported by studies on their ability to inhibit inflammation, support the growth of beneficial bacteria, and enhance cell metabolism [95,96,97,98,99,100,101,102,103].

Mineral-based nanoparticles (especially AgNPs) have the greatest pharmacological targets in the wound healing process, suggesting their great therapeutic capabilities from among the many techniques utilized to produce nano formulas of phytochemicals [48,49,104]. The significance of natural chemicals as alternatives for treating different wounds has been verified by the presented findings of numerous studies, and the effectiveness of nanotechnology in improving the efficiency of various medicines has been improved because of improvements in targeted treatment and bioavailability as well as increased stability. Nanostructure approaches to natural wound healing agents have received a great deal of interest; however, to assess the intracellular targets implicated in the wound-healing benefits of natural nanomedicine, further pharmacological studies are required in order to validate the safety and effectiveness of nano-formulations based on natural ingredients in the treatment of wounds, and well-designed clinical studies are also required [105].

Electrospun nanofibers containing bioactive materials can be developed, and it have been widely used in various fields. There are many uses for electrospun fibers containing bioactive materials in a variety of applications. Therefore, in the next section, a detailed overview will be provided of several areas using electrospun fibers in multiple applications, including biomedical and pharmaceutical textiles.

### 6.1. Anticancer Nanofibers:

Until now, cytotoxic anticancer agents have been the most effective means of controlling tumour growth. However, this method still suffers from off-target toxic effects on normal tissue. This increases the need to focus on localized delivery of cytotoxic anticancer agents [106]. Implementation of a biocompatible system after tumour surgery can get rid of remaining residuals and control tumour relapse. Nanofiber loaded with anticancer agents can act as a novel effective delivery system for such use due to its large surface area, porosity, and tensile flexibility [48,49,106].

Chemotherapeutics which can be loaded into nanofibers include cisplatin, paclitaxel, fluorouracil, and curcumin. In addition, metal nanoparticles such as carbon nanotubes, silver nanoparticles and zinc oxide can act as robust anticancer agents [107,108,109,110]. Combination therapies are rising because of cancer resistance to single agent. A group has loaded PVA nanofiber with folic acid conjugated PCL/PEG/DOX, which enhanced DOX uptake by cancer cells [90,91]. Yun et al. have demonstrated an electro-stimulated nanofiber made of PVA/PAA/carbon nanotubes that completely controlled tumour growth, thanks to polymer swelling leading to the instant release of treatment in response to an electric charge [89]. Liu et al. synthesized a calcium titanate nanomaterial into PAA nanofiber [90]. Wei et al. have combined ZnO with DOX into PLGA/gelatine nanofiber in order to completely control cancer [91]. Anticancer nanofibers are still until investigation in preclinical and clinical studies, and are promising as a drug delivery system.

### 6.2. Antibacterial Wound Dressings

Wound healing is considered a complex physiological route and it is identified with a coordinated reaction of various cell sorts and development elements for accomplishing tissue recovery. Wounds can be characterized as any damage to the skin caused by an injury. Wounds are caused by an assortment of superficial or chronic injuries, for example, injury, surgery, pressure and so forth. Healing is a long and complex procedure through which the skin or body tissue repairs the dead or infected skin or tissue in order to help heal the wounds. The required time for the healing process depends on the area as well as the location and size of the wound [48,49,111,112,113,114,115].

Open wounds are exposed to contamination and bacteria, resulting in an extended inflammatory stage and enhanced expression of metalloproteinase, which is involved in the decomposition of ECM components and prevents the producing of new granulation tissue. When wound dressings are used to cover an infected skin wound, the antimicrobial dressing works as a protective barrier to prevent the prevalence of all anaerobic bacteria into the wound, and subsequently destroys harmful microorganisms. Additionally, the antimicrobial dressing improves the healing of wounds by stimulating the immune response and migration of fibroblasts and keratinocytes. The antimicrobial polymeric dressing, designed to act as a protective impediment that safeguard the wound from harmful microbial overrun, support fibroblast migration and differentiation, and absorb exudates [48,49,116].

Conventional dressings are utilized to shield wounds from further damage during the recuperating procedure. The ideal wound dressing should: (1) have the ability to absorb excess fluids and drain pus accumulating in the wound area; (2) keep the surrounding wound environment humid; (3) work as a barrier to prevent bacteria growing in the wound dressing; (4) be sterile, non-allergic and non-toxic; (5) be easy to change and painless on removal; (6) be effective and inexpensive [117,118,119]. Keeping in mind the final aim of encouraging healing, biomaterial dressings are frequently utilized. The perfect features of a wound dressing ought to empower the local extracellular network of skin and look after hemostasis, oxygen pervasion, and epithelization (by discharging organic specialists to the injuries), as well as repress the intrusion of exogenous microorganisms or outside elements [118,119,120,121,122].

Electrospun nanofiber mats are considered a great hopeful in wound dressing production because they have a moderately bigger surface area contrasted with other customary dressings. This one-of-a-kind property empowers them to retain, as much as possible, liquids and pus from the wound site. Additionally, several antimicrobial agents and drugs can be incorporated into the nanofibers during electrospinning. In this case, they can be used widely to aid in wound healing in various surgeries, medical interventions, and hospitals. For example, it was reported previously by our group [21] that fabrication of electrospun “carboxymethyl chitosan” “polyethylene oxide” nanofibers embedded AgNPs resulted in antimicrobial wound dressing nanofiber comprising AgNPs that could be considered a good candidate for healing of different kinds of wounds and ulcers [27,48,49,123,124].

Also, El-Aassar et al. [49] synthesized electrospun nanofibers fabricated from polyvinyl alcohol/Pluronic F127/polyethyleneimine (PVA–Plu–PEI) nanofiber, which were loaded with various ratios of titanium dioxide nanoparticles (TiO_2_ NPs) with particle sizes between 4 and 20 nm. They examined the effect of the antimicrobial activity of the PVA–Plur–PEI/TiO_2_ composite nanofiber by zone inhibition against tested bacteria. The results revealed that the antibacterial activity exhibited enhanced the antimicrobial efficiency of the PVA–Plur–PEI/TiO_2_ over the PVA-Plur-PEI nanofibers against all the examined bacterial strains, especially, Gram-negative bacteria.

The well-known advantages of utilizing antimicrobial nanofiber mats loaded with AgNPs, TiO_2_, and Zinc peroxide (ZnO_2_) in the healthcare sector can be described in terms of (i) high effectiveness, i.e., high content of surface, more active, (ii) minimal influence on physical and mechanical properties, (iii) minimal or no use of toxic chemicals, (iv) lower energy consumption and cost, and (v) lower environmental impact.

Polymers with antimicrobial activity can be divided into two categories. The first category includes all polymers which exhibit antibacterial properties or intrinsic antimicrobial activity (antimicrobial polymers); these can be applied in a variety of applications directly. The second category contains inactive polymers that include antimicrobial agents for improved antimicrobial performance and controlling the release of antimicrobial agents over time [96]. Antimicrobial polymers possess an inherent chemical composition that can improve the efficacy of antibacterial activity. This antimicrobial activity can be attributed to two main causes: (1) a polymer structure with quaternary nitrogen groups; and (2) ε-poly-L-lysine(ε-PL) and halamines which can act as a backbone to improve the resistance of existing microbes [97]. A biopolymer of chitosan and chitosan derivatives such as carboxymethyl chitosan and quaternized chitosan has a broad spectrum of antimicrobial activity against bacteria, fungi, and yeasts [98]. Many studies have investigated electrospun NFs with antimicrobial activity produced using biopolymers such as chitosan and its derivatives, or by blending chitosan with other polymers such as polyvinyl alcohol (PVA) [99] and poly (ethylene oxide) (PEO) [100,101]. In order to confer antimicrobial properties to NFs, different antimicrobial agents can be incorporated into the nanofibrous structure [125,126,127,128,129,130,131,132,133,134,135,136]. Antimicrobial agents such as antibiotics (e.g., tetracycline, ciprofloxacin, gentamicin etc.), metal nanoparticles (e.g., zinc oxide (ZnO), titanium dioxide (TiO_2_) and silver (Ag) nanoparticles) and natural products (e.g., honey, Aloe Vera) or volatile oils (e.g., cinnamon oil, etc.) have been incorporated into NFs for biomedical applications.

Nanofibers incorporating antibiotics are promising materials in pharmaceutical and tissue engineering applications [98,137,138,139,140]. These nanofibrous structures may be possible to use with embedded drugs and to load polymeric microspheres, micelles, or liposomes. To minimize the risk of antimicrobial resistance, non-antibiotic antimicrobial-based NFs have been developed. Examples of non-antibiotic antimicrobial agents include metal and metal oxide nanoparticles, e.g., ZnO NPs [102], as well as volatile oils such as cinnamon oil [116,117,118].

#### 6.2.1. Applications of Essential Oil-Loaded Nanofibers

Essential oils (EOs), strong natural and medicinal plant products, are known for their medicinal value. They have been utilized as perfumes, flavours for foods and beverages, and to heal both mind and body. In ancient Egypt, EOs were extracted by infusion. The Romans and Greeks used the distillation process so that aromatic plants could provide additional value. Moreover, the study of the nature and components of essential oils was later expanded by the Europeans who developed this science [104].Generally, essential oils are extracted by distillation from aromatic plants as crude plant extracts and mixed with various chemical compounds, such as phenolic compounds (eugenol, and carvacrol), alcohols, and ethers. The mechanism of action of EOs is dependent on the disturbance of bacterial cell lipids due to their hydrophobic nature. The cell membrane is destroyed, and the ions penetrate inside to release other cellular components, finally resulting in breakdown of the proton pump and ultimately leading to cell death. EOs can be encapsulated in nanostructures such as nanoparticles [105] and films [106] for enhanced efficacy. Various EOs, including cinnamon, candeia, tea tree, cloves, peppermint, thyme, and lavender, have been incorporated into electrospun nanofibers [107]. Cinnamon essential oil from the cinnamon tree (CEO)exhibits diverse biological functions, including anti-inflammatory, antioxidant, antimicrobial, and anti-diabetic effects. Cinnamon is rich in EOs and tannins, which inhibit microbial growth. The chemical constituents of cinnamon include volatile oils, e.g., eugenol, cinnamic acid, cinnamaldehyde, mucilage, diterpenes, and proanthocyanidins [108]. Eugenol is widely utilized and well known for its medicinal properties [109]. It has a strong antibacterial effect against oral bacteria linked to tooth decay and gum disease, and is effective against a large number of other bacteria and viruses without the side effects. The wound healing activity of cinnamon was confirmed in order to determine the impact of its anti-inflammatory, anti-microbial and anti-oxidant effects on the healing of wounds [14].

Incorporation of bioactive compounds and bioactive agents into nanofibers has been used to create new and effective dressings used to increase healing efficiency and repair wounds. The desired release profile of the scented oils can be accomplished by loading the EO in a particulate carrier as a potential controlled delivery system [141,142,143]. Encapsulation of EOs in hydrophilic polymers (e.g., PVA and PEO) led to the lowering of their volatility, which is a significant feature required for longer shelf-life period and enhanced stability of the product [138]

Hulya et al. have successfully produced antibacterial Polyvinyl pyrrolidone/cinnamon essential oil (PVP/CEO) NFs by oil-in-water emulsion electrospinning, using different concentrations of cinnamon oil (1%, 2%, 3%, 4% and 5%) and (Cremophor RH 40) as an emulsifier. The nanofiber efficacy was determined in antimicrobial assays via the disk diffusion method using Staphylococcus aureus, Escherichia coli, Candida albicans, and Pseudomonas aeruginosa. The largest inhibition zone diameters were produced with PVP/5% cinnamon oil for *S. aureus* (11mm) and with PVP/2%cinnamon oil for *E. coli* (8mm). For the PVP/2% cinnamon oil and PVP/3% cinnamon oil the same held true for the diameter of the zone of inhibition, and for PVP/4% cinnamon oil it was almost the same (*C. albicans* 9 mm). Moreover, antibacterial activity was observed to increase with the size of the nanofibrous material [144].

The main goals of wound care are the rapid healing of skin layers and prevention of infections that might compromise the process of wound healing [132,133,134,135,136,137,138,139]. The major negative impact on the healing process is when wounds are colonized by microorganisms, which delays the entire wound healing process. In a study conducted by I. Liakos and co-workers, they used EOs as natural antimicrobial agents encapsulated in cellulose-based nanofibers. They demonstrated the production of composite fibers encapsulating three different EOs (cinnamon, lemongrass, and peppermint) with concentrations (1 or 5%) *v/v*. The NF scaffolds inhibited the expansion of Escherichia coli even when the smallest quantity of essential oils was used, and no signs of cytotoxicity were observed; by using two cell lines, the biocompatibility of the NF scaffold was assessed for successful wound healing and regeneration of cutaneous tissue (immortalized fibroblasts and normal human keratinocytes). The produced skin wound dressings were promising candidates for biomedical application and treating several types of wounds [141,142,143,144,145,146,147,148,149,150,151,152,153,154,155,156,157,158,159,160,161,162,163,164,165,166,167,168].

Katrina A. Rieger and Jessica D. Schiffman successfully fabricated electrospun chitosan/PEO NFs containing cinnamon oil with an average diameter of 38–55 nm. Chitosan and PEO were blended at a volume ratio (1:1) in 5 *w/v* % aqueous solutions of acetic acid containing diverse concentrations of cinnamon oil (0.5 and 5.0 *v/v* %). The chitosan/PEO nanofibers were cross-linked via contact to vapor of glutaraldehyde to improve the chemical and hydrolysis stability. The nanofiber mats were examined against *P. aeruginosa* and showed an inactivation rate in the bacteria of 76% and 50% for the mats with and without 5% cinnamon oil, respectively. The chitosan/PEO/cinnamon oil composite NFs reduced *E. coli* activity after contact with the bacteria within 30 min, and its lost viability after 180 min was higher than 99% [146].

El-Aassar et al. synthesized an HA/PVA/PEO blend using the electrospinning technique. These nanofibers were incorporating with ZnO nanoparticles/cinnamon essential oil (CEO) [26]. The results showed that the ZnO NPs/CEO embedded within the HA/PVA/PEO nanofibers inhibited the growth of Staphylococcus aureus (*S. aureus*), and HA/PVA/PEO-ZnO NPs/CEO nanofibers showed that the ZnO NPs/CEO loaded nanofibers exhibited enhanced antibacterial effect, healing speed, and quality of skin restoration compared to HA/PVA/PEO nanofibers.

Clove EO is an essential oil obtained from the buds of the clove plant and leaves of Syzygiumaromatic [113]. Clove EO is used in medical applications as a therapeutic compound, pharmaceutical, and also as a flavouring in the food industry. Clove oil and its main component, eugenol, may have benefits for the treatment of dental caries and periodontal diseases, and as an anti-inflammatory, antioxidant and anti-microbial which works on the acceleration of wound repair and even fighting cancer [114].

Clove EO has been loaded and encapsulated with a variety of polymers such as sodium alginate/PVA [115], Poly(ε-Caprolactone)/Gelatine [116], and bacterial cellulose [117]. Unalan et al. [116] prepared antibacterial electrospun nanofiber using poly ε-caprolactone/Gelatine (PCL/GEL) with clove EO via the electrospinning technique. First, the Clove EO (CLV) was incorporated in 4.8%, *w/v* form PCL/GEL solutions. The results showed that the CLV-loaded PCL-GEL nanofiber showed excellent antibacterial activity against *S. aureus* and *E. coli*.

#### 6.2.2. Applications of Metal Nanoparticle-Loaded Nanofibers

Metals can be produced in nano-sized particles that display interesting antimicrobial properties against diverse pathogens due to their low toxicity, extremely large specific surface area, long-term action, and extreme stability.

Metal nanoparticles have increasingly attractive properties for use as effective antimicrobial materials due to the large, rapid and widespread development of antimicrobial resistance [118]. There are many examples of metallic nanoparticles (NPs) such as copper, gold, silver, zinc, titanium and magnesium that function as antimicrobial agent’s different types of bacteria, viruses, and other microorganisms. Incorporation of these metal nanoparticles into electrospun NFs would provide a potential platform in many applications, particularly in the field of medical applications [119].

Among metal nanoparticles, ZnO NPs have emerged a promising agent in biomedicine, especially in the antibacterial and anticancer fields, which involve the potent ability of ZnO NPs to release zinc ions, causing excessive generation of ROS and inducing cell damage [120]. Furthermore, Zinc oxide NPs show premium luminescent properties and can thus be one of the main candidates for bio-imaging. ZnO NPs have been successfully applied as embedded antimicrobial agents in medical applications such as wound healing, packaging, and tissue engineering applications. Electrospun polymer-based scaffolds embedded with ZnO NPs have shown improved cell reproduction and wound healing in addition to their anti-bacterial properties [121,122].

Zinc oxide NPs have promising biomedical potential, exemplified in their antimicrobial activities and anticancer properties. ZnO NPs have an inherent ability to produce ROS and cause apoptosis; in addition, ZnO NPs can be used as drug carriers and increase potency at the target sites, thus reducing unwanted toxicity and off-target effects and broadening the range of synergistic effects [123].

One of the serious complications of diabetes is non-healing wounds, very slow wound closure, and a high incidence of infection. Rashid Ahmed and co-workers hypothesized that NF mats composed of a blend of chitosan, polyvinyl alcohol (PVA) and Zinc oxide (ZnO) could be an effective agent for rapid healing of the wounds of diabetics because of the wound healing activity of chitosan-PVA NFs and antibacterial properties of ZnO. NF mats were evaluated for their antibacterial and antioxidant activities and in vivo wound healing in rabbit models. The chitosan/PVA and chitosan/PVA/ZnO nanofiber showed higher antioxidant properties, and within a 12-day period wounds were completely healed. Therefore, it was concluded that chitosan/PVA/ZnO nanofibers can be used as a useful dressing for diabetic wounds [124].

Ayesha Khalid and co-workers have impregnated zinc oxide NPs (1%) into bacterial cellulose (BC) NF sheets [125]. The impregnation of NPs into bacterial cellulose was confirmed by structural characterization, and the antimicrobial activity of BC-ZnO composite nanofiber was tested against common burn wound infection pathogens. BC-ZnO composite nanofiber showed antimicrobial activity against three strains of Gram-negative (*E. coli* (90%), C. fundi (90.9%) and *P. aeruginosa* (87.4%)) and Gram-positive (*S. aureus* (94.3%)) strains. The results successfully demonstrated composite nanofiber *invivo* burn wound healing in a BALBc mouse model, as well as tissue regeneration.

Kyung Lee and Seungsin Lee estimated the antimicrobial potential of PVA/ZnO (11%:3%) composite nanofibers to improve the effectiveness of antibacterial nanofiber in the inhibition of bacteria by carrying out wear trial experiments to verify the feasibility of composite nanofibers in real use cases. A very thin layer of a ZnO/PVA was applied directly onto nonwoven layers as a carrier for polypropylene webs, the nonwoven layer acts as the substrate for thin composite nanofibers layer. Eight players participated in a basketball game for two hours wearing the prototype shoes that had a very thin layer of ZnO/PVA nanofibers on the sole of the shoe. The results showed a reduction in Staphylococcus aureus of 80% up to 22 days in wear trials, where the ZnO/PVA thin nanofibers were applied to shoe insoles. In the in vitro study, the results showed a 99.9% reduction in Staphylococcus aureus. In addition, the ZnO/PVA nanofibers in another group of shoe insoles were maintained at a constant temperature and moisture level after the third wear experiment; the results showed a reduction of 66.0% and 86.6% of Staphylococcus aureus after 15 and 22 days, respectively [126].

The genuine capacity of the antibacterial mechanism of AgNPs is accompanied by the connection amongst Ag and the “thiol” groups found in both cells of bacteria and/or fungi. While the precise mechanism is still unidentified, it has been concluded that upon contact with AgNPs, structural variations in bacterial and fungal cells are displayed. AgNPs have favorable antibacterial and antifungal activity compared to regular silver, which is attributed to a relatively enormous surface area that tolerates greater contact with bacteria and fungus cells. Moreover, AgNPs not only invades the cell membrane but also enters the cells of both bacteria and fungus, fixing to the membrane cell wall and hindering the process of respiration [127].

The absorption of phosphate and the release of mannitol, succinate, proline and glutamine is dampened by the presence of silver in the case of Escherichia coli. Consequently, AgNPs effectively inhibit the growth of several bacteria and fungi, and they are appropriate for controlling different antibacterial systems [128,129]. Scientists have also compared the biological efficacy of nanofibers embedded with AgNPs and other regularly known polymers in term of wound healing. All results conclude that AgNP loaded polymers such as (PVA-Ag) retain the best antibacterial activity and the uppermost healing potential, as seen in Table 2 [130]. As it shows, PVA comprising AgNPs display superlative healing efficiency. Both wound depth and wound area become smaller compared to nine other dressing membranes (cotton gauze, cross-linked PVA, “wool protein-coated PVA, wool protein/PVA co-electrospun nanofibers, PCL (poly-caprolactone), PCL-p-coated (wool protein-coated), PAN, PAN-PEU (polyurathane), and no dressing (control) after 16 days. This specifies that the wounds cured with PVA mats embedded AgNPs recovered better and faster, with stronger antimicrobial activity.

As was previously reported by our group [131], fabrication of electrospun nanofiber comprising Polygalacturonic/Hyaluronic acid embedded silver nanoparticles resulted in an antimicrobial. The nanofiber mats with embedded silver nanoparticles were considered a good candidate for antioxidant and anti-inflammatory action and accelerated wound healing for different kinds of wounds and ulcers.

## 7. Conclusions and Future Outlooks

Nanofiber nanocomposite materials are used in diverse applications, especially for biomedical applications including drug delivery, anticancer, and wound healing, due to their unique advantages. The papers reported in this review have shown that bioactive compounds such as essential oils, herbal bioactive components, plant extracts, and metallic nanoparticles containing polymer nanocomposites have all drawn tremendous interest for applications in biomedicine and cancer treatment. The addition of bioactive components has been shown to improve biocompatibility and non-toxicity and to enhance bioactivity, full repair, and regeneration.

Electrospinning remains a slow and time-consuming technique that is limited by the properties of the materials used; highly viscous materials can cause needle clogging or poor mechanical properties in the generated nanofibers. In the future, combining bioactive components with natural and synthetic polymers to produce composites for production in combination with the performance of electrospun nanofiber loaded with bioactive components; represents an efficient strategy for the fabrication of biodegradable materials able to rapidly integrate with wounds, creating exciting opportunities to promote biological systems and tissue regeneration. Indeed, a full understanding the current devoted efforts by researchers will allow the optimization of the production of electrospun nanofiber loaded with bioactive components and designs, and the advanced technologies for using it in industrial applications.

## Figures and Tables

**Table 1:** Comparison between different methods of nanofiber production.

Technique	Process	Scalability	Control over Nanofiber Dimension	Advantages	Disadvantages
**Drawing** **[12]**	Laboratory	No	No	No need for special equipment	Discontinuous process and not scalable
**Template** **Synthesis** **[13]**	Laboratory	No	Yes	Nanofibers with various diameters can be obtained by using various templates	Non-uniform size distribution. Organic solvents required Full difficulty of interconnectivity
**Phase** **Separation** **[14]**	Laboratory	No	No	Minimum equipment requirement for operations Used in fabrication of broad range of biomaterial Ideally suited to direct fabrication	Organic solvents present or necessary Skinning effect on the scaffold morphology
**Self-Assembly** **[15,16]**	Laboratory	No	No	Usually performed in water Bioactive functionality	Manufacture process is very complex Cost of manufacturing is prohibitively high Low mechanical properties
**Electrospinning** **[9,17,18]**	Laboratory and industry	Yes	Yes	Cost effective Wide range of different polymers are used Excellent biocompatibility and biodegradable materials Extensive surface area and large pores	Organic solvent often required Instability of jet

**Table 2:** Natural polymers and synthetic polymers for nanofiber fabrication.

Polymer Material		Solvent*	Biodegradability	Advantages	Disadvantages	Ref
**Natural Polymers**	Chitosan	H_2_O/CH_3_COOH	Fast-biodegradable	- Natural origin - Biocompatibility - Biodegradable - Fewer side effects and non-toxic - Easily available and inexpensive-Bioactivity	- Poor mechanical properties - Microbial contamination - High degree of variability in natural materials extracted from animal sources - Structurally complex - Extraction process very complicated and expensive - Difficult to processing	[21]
	Cellulose	CH_3_COOH	Fast-biodegradable			[8]
	Silk	HCOOH	Slow-biodegradable			[22]
	Gelatin	HFIP	Slow-biodegradable			[23]
	Collagen	(CF_3_)_2_CHOH	Slow-biodegradable			[24]
	Alginate	H_2_O	Fast-biodegradable			[25]
	Hyaluronic acid	DMF/H2O	Fast-biodegradable			[26]
	Starch	H_2_O	Fast-biodegradable			[27]
	Tragacanth gum	HCOOH	Slow-biodegradable			[28]
**Synthetic Polymers**	Poly(vinyl alcohol)	H_2_O	Fast-biodegradable	- Biocompatibility - Better mechanical properties - Easily produced and inexpensive	- Toxic - Poor biodegradability - Water solubility - Synthetic process is very complicated and expensive	[29]
	Poly(ethylene glycol)	H_2_O	Fast-biodegradable			[30]
	Poly(ethylene oxide)	H_2_O	Fast-biodegradable			[31]
	Poly(lactic acid) (PLA)	THF/DMF	Slow-biodegradable			[32]
	Poly(ε-caprolactone)	CHCl_3_/CH_2_Cl_2_	Slow-biodegradable			[33]
	Poly(lactic-glycolic acid)	THF/DMF	Slow-biodegradable			[34]
	Poly(urethane)	THF/DMF	non-Biodegradable			[35]
	Poly(ethylene vinyl acetate)	CHCl_3_	non-Biodegradable			[36]

Polymers, w; * (CH_3_COOH) Acetic acid, (HCOOH) Formic acid,(HFIP)1,1,1,3,3,3-hexafluoro-2-propanol, (CF_3_)_2_CHOH) 1,1,1,3,3,3-Hexafluoro-2-propanol, N,N-Dimethylformamide (DMF), (CH_2_Cl_2_ ) dichloromethane, (CHCl_3_) Chloroform, and(THF) Tetrahydro furan.
